# Enhancing glucose flux into sweat by increasing paracellular permeability of the sweat gland

**DOI:** 10.1371/journal.pone.0200009

**Published:** 2018-07-16

**Authors:** Andrew Jajack, Michael Brothers, Gerald Kasting, Jason Heikenfeld

**Affiliations:** 1 Department of Biomedical Engineering, University of Cincinnati, Cincinnati, Ohio, United States of America; 2 UES, Incorporated, Dayton, Ohio, United States of America; 3 711th Human Performance Wing, Air Force Research Laboratory, Wright-Patterson Air Force Base, Ohio, United States of America; 4 James L. Winkle College of Pharmacy, University of Cincinnati, Cincinnati, Ohio, United States of America; 5 Department of Electrical Engineering and Computing Systems, University of Cincinnati, Cincinnati, Ohio, United States of America; 6 Eccrine Systems, Incorporated, Cincinnati, Ohio, United States of America; Hungarian Academy of Sciences, HUNGARY

## Abstract

Non-invasive wearable biosensors provide real-time, continuous, and actionable health information. However, difficulties detecting diluted biomarkers in excreted biofluids limit practical applications. Most biomarkers of interest are transported paracellularly into excreted biofluids from biomarker-rich blood and interstitial fluid during normal modulation of cellular tight junctions. Calcium chelators are reversible tight junction modulators that have been shown to increase absorption across the intestinal epithelium. However, calcium chelators have not yet been shown to improve the extraction of biomarkers. Here we show that for glucose, a paracellularly transported biomarker, the flux into sweat can be increased by >10x using citrate, a calcium chelator, in combination with electroosmosis. Our results demonstrate a method of increasing glucose flux through the sweat gland epithelium, thereby increasing the concentration in sweat. Future work should examine if this method enhances flux for other paracellularly transported biomarkers to make it possible to detect more biomarkers with currently available biosensors.

## Introduction

Sweat is separated from the surrounding biomarker-rich blood and ISF by a one- to two-cell thick epithelium [1]. This leaves only two routes of entry for biomarkers—*transcellular* (through the cells) or *paracellular* (between the cells). For most analytes, the cellular lipid bilayers form a barrier for *transcellular* diffusion, while the tight junctions between the cells form a barrier for *paracellular* diffusion. Moderately hydrophobic and small molecules (e.g., cortisol, ethanol) can pass through the lipid bilayer and enter sweat at blood-level concentrations. However, larger, uncharged, polar molecules (e.g., glucose) and charged molecules (e.g., ions or proteins) must move through the space between the cells. Even though this space is 10’s of nanometers wide [2], tight junctions pinch neighboring cell membranes together to form a seal that makes it difficult for anything but small (<100 Da) ions to pass. For example, proteins are ~1000x more dilute in sweat (<nM) compared to blood; glucose is ~100x (<mM) more dilute [3–5]. Therefore, many biomarkers that would be useful to monitor are diluted in sweat to concentrations below what can be detected by available biosensors.

**Fig 1:**
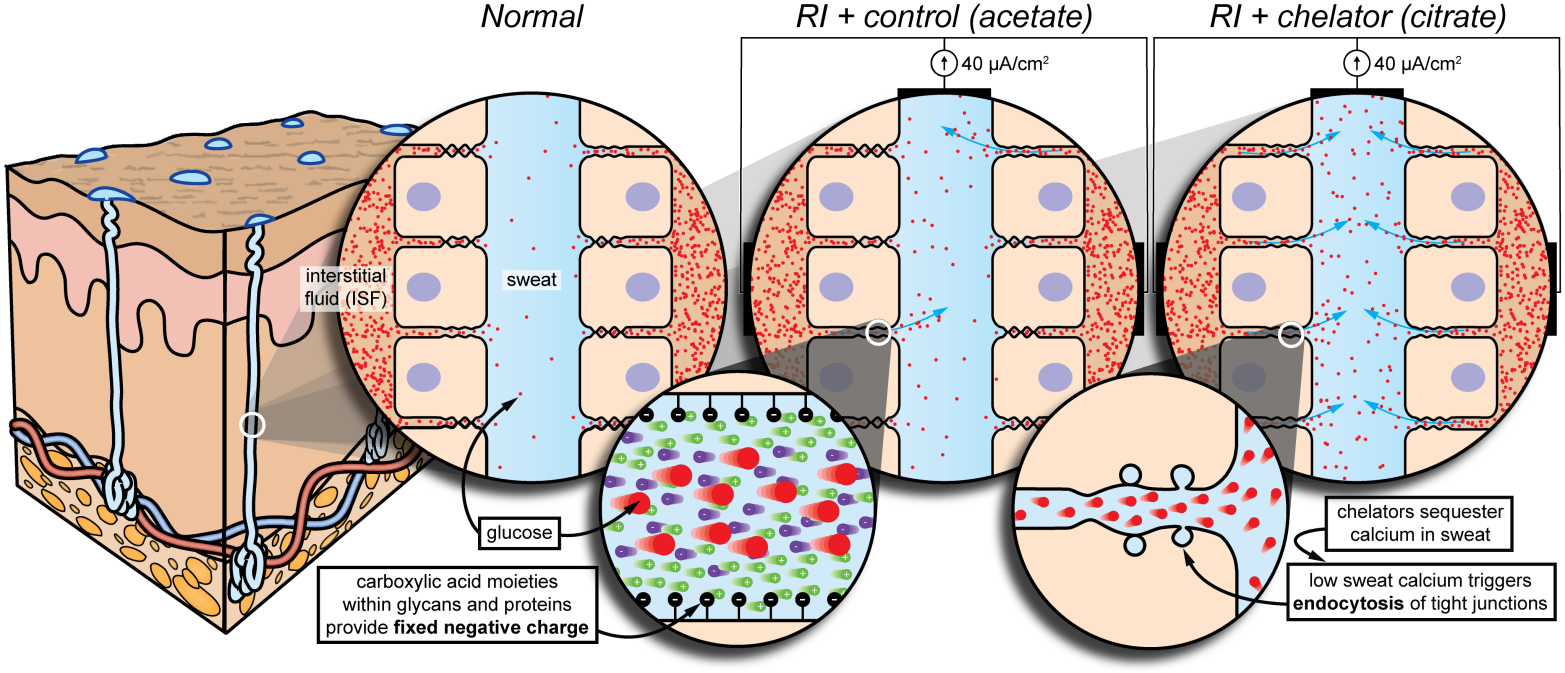
Conceptual model for glucose flux under various conditions: Normal, electroosmosis, and chelation with electroosmosis. **Normal**. Tight junctions undergo constant and spontaneous remodeling. While this can cause direct aqueous paths to form momentarily, the majority of glucose likely diffuses from ISF to sweat between opening and closing compartments that fluctuate between the tight junctional strands. **RI + control (acetate)**. Electroosmosis (enlarged) is the flow of fluid resulting from an applied electric field in the presence of an electrical double layer. The fixed negative charges needed to establish the double layer are due to the carboxylic acid moieties within glycans and proteins on the cell surface. Electroosmosis brings in ISF through the paracellular pathway. **RI + chelator (citrate)**. Chelation triggers reversible endocytosis of tight junctions (enlarged), producing many more direct aqueous paths, which close upon reintroduction of calcium. Therefore, we expect electroosmosis with chelation to facilitate much higher glucose flux compared to electroosmosis alone. Skin model has been adapted [1].

Paracellular permeability of small biomarkers relies on the dynamic nature of tight junctions. Tight junctions make up a flexible network of strands that continuously break, reseal, and branch [6,7]. While this reshaping can cause direct aqueous paths to form momentarily, most biomarkers likely diffuse from ISF to sweat between opening and closing compartments that fluctuate between the strands [6,7], creating a tortuous path (Fig 1).

Several groups have artificially modulated the permeability of tight junctions in the intestinal epithelium to increase drug absorption [8–12]. Since tight junctions are calcium-sensitive, calcium chelators are effective tight junction modulators [12]. Chelators sequester calcium ions and trigger endocytosis of tight junctional proteins [9,13–15], opening up the paracellular pathway [16]. We postulate that this method of increasing paracellular permeability for intestinal drug delivery could also be used to improve the extraction of biomarkers in other epithelia such as the sweat gland epithelium.

However, simply applying a chelator topically would not be effective, as the rate of diffusion into the gland would not be sufficient to counter the advective flow of sweat out of the gland. Most chelators are negatively charged in the pH range of sweat, and so instead, iontophoresis can be used to selectively drive chelators down into the lumen of the sweat gland. An additional benefit, the reverse iontophoresis (RI) needed to drive a negatively-charged chelator also happens to induce electroosmotic flow of the surrounding interstitial fluid into sweat through the paracellular pathway [17] (Fig 1). The negatively-charged carboxylic acid moieties within glycans and proteins on the cell surface provide the electrical double layer needed for electroosmosis. This electroosmosis has been previously used for diluted glucose extraction through the skin in an FDA-approved commercially-available product, the GlucoWatch Biographer [18–20].

As depicted in Fig 1, we speculate that reverse iontophoresis (RI) triggers electroosmosis through the paracellular pathway. We also speculate that chelation triggers endocytosis of tight junctions in the sweat gland, producing many more direct aqueous paths leading to lesser dilution of the ISF. Therefore, we expect RI in the presence of chelators will facilitate much higher flux of biomarker-rich ISF into sweat compared to RI without chelators. Furthermore, because sweating provides an outward advective flow, the constant and 10X larger current (0.5 mA/cm^2^) of the GlucoWatch Biographer is not required.

Here, we examine sweat glucose flux under various conditions: normal, RI without a chelator, and RI with a chelator (Fig 1). We use a skin-safe [21] calcium chelator, citrate (lemon/lime juice), as our permeability enhancer [12], and acetate as our control. This study is the first to use a permeability enhancer for extraction rather than drug delivery and the first to combine permeability enhancement with electroosmosis. Our proof of principal, demonstrated by the marked increase in glucose flux, shows promise for further optimization of paracellular permeability enhancement with electroosmosis as a viable option for overcoming detection challenges in sweat biosensing. This work could also prove useful for enhanced transdermal drug delivery.

## Materials and methods

### Custom testing device

All tests are performed using a custom testing device—printed using a stereolithographic (SLA) 3D printer (Formlabs Form 2) in UV-curable plastic resin—consisting of a base that adheres to the skin and a set of removable attachments that lock into the base (S1 Fig). The base has an adhesive-covered (3M 1577) flange that firmly adheres to a ring of skin surrounding the testing area (circular region with 28 mm diameter). This, combined with a Velcro strap, locks the testing area in place and prevents it from slipping as the participant moves. The attachments mate with the base using a twist-lock mechanism. Attachments include those for stimulating sweat production, collecting sweat with or without RI, and measuring sweat rate. Before attaching the device, participants are instructed to wash the testing area with soap and water. The area is then cleaned with isopropyl alcohol (Sigma-Aldrich), rinsed with deionized (DI) water, and then dried thoroughly by blotting with wipes (Kimwipes, Kimtech Science) and evaporating with compressed nitrogen.

### Sweat generation method

Carbachol—a slowly-metabolized cholinergic agent—has been shown to provide localized sweat stimulation when delivered iontophoretically (S2 Fig) [22]. Carbachol was sourced in bulk form (active pharmaceutical agent) from a local compounding pharmacy. A carbachol-containing gel disk (1% carbachol, 3% agarose, 6.1 cm^2^ x 6 mm thick) is placed into the testing device, and an attachment with a conductive-carbon layer locks into the device, pressing the disk against the skin (S3 Fig). A disposable counter electrode (commonly used for TENS stimulation) is then placed on the adjacent skin. A lab-grade potentiostat (Gamry 600) drives the carbachol into the skin by applying a constant current density of 0.02 mA/cm^2^ for 10 minutes. It takes about 15–30 minutes to produce an adequate sweat response in most people. After reaching a peak sweat rate, sweat rate begins to slowly decline over hours or even days [22].

Wescor’s FDA-approved Nanoduct device uses a current density of 0.25 mA/cm^2^ applied over 2.5 minutes. While this produces adequate sweat generation rates, we were concerned that any excess in applied current could trigger electroporation. To avoid this, we reduced the current by a factor of ~10x (0.02 mA/cm^2^) and extended the time to maintain a similar dose. Since this produced adequate sweat generation rates, no further optimization was performed.

### Avoiding sweat contamination

Sweat needs to be collected on the skin, but the skin can contaminate samples by harboring microbes, accumulating analytes, and sloughing off cells. Both the density and diversity of the microflora (bacteria, fungi, and viruses) differ based on the region of the body and can differ widely between people and even within the same person over time [23,24]. Estimates of the density of bacteria found on the skin are as high as 10 billion/cm^2^ [25]. Bacteria can consume analytes such as energy sources like glucose and secrete analytes such as proteins or cellular waste products. These alterations of systemic levels of analytes by the microflora pose a challenge to accurate measurement of biomarker concentrations. In addition, sweat minerals have been shown to accumulate in the superficial layers of the epidermis and possibly in the sweat duct itself prior to sweating events [26,27]. It can be assumed that similar accumulation may occur with other biomarkers, lowering the time resolution of the sweat sample. Finally, the skin surface is constantly being coated with proteases which aid in the shedding of dead skin cells and a mixture of triglycerides, wax esters, squalene, and metabolites from sebaceous glands may cause a problem for protein detection [28,29]. Skin surface contaminates become an even larger issue for small samples. These issues can be avoided by preventing sweat from contacting the epidermis by coating the skin with an occluding layer of petroleum jelly or oil [30,31] (S4 Fig).

### Sweat collection method

A thin layer of petroleum jelly is applied to the testing area using a sterile cotton applicator. Then, the testing area is cleaned with isopropyl alcohol, rinsed with DI water, and dried thoroughly. Any excess petroleum jelly is removed by blotting with a wipe. To allow for almost complete sample recovery, absorbent disks are made of a non-woven hydrophilic mesh (Sefar Inc. Nitex 03-110/47) with low non-specific binding to analytes. Disks are laser cut (VLS3.50, Universal Laser Systems) to a diameter of 28 mm. An absorbent disk is placed within the base of the device and kept pressed against the skin using an attachment that locks into the base (S4 Fig). At the end of the collection period, the absorbent disk is removed. The collected sweat is then extracted by centrifugation and stored in a -20°C freezer.

### Sweat glucose measurements

Sweat glucose is determined using an enzymatic glucose assay (Amplex Red Glucose Assay, Thermo Fisher Scientific) read via a microplate reader (Synergy H1, BioTek). Before analysis, sweat samples are thawed to room temperature and are diluted by at least two-fold using the buffer (pH 7.4) provided with the assay to mitigate the effects of pH on enzyme activity.

### Blood glucose measurements

Blood glucose is measured using a fingerstick glucometer. Before each measurement, participants are instructed to wash their hands with soap and water. The finger to be tested is cleaned with isopropyl alcohol, rinsed with water, and then dried thoroughly. Blood is tested using Accu-Chek Aviva Plus test strips (Roche Diagnostics), which had the best performance when compared to other commercially available strips [32].

### Sweat rate measurements

Sweat rate is determined gravimetrically (S5 Fig). Absorbent disks (TX609 TichniCloth, Texwipe) are laser cut to a diameter of 28 mm and individually weighed on an analytical balance (ViBRA Shinko Denshi HT224R). Then, the surface of the skin is thoroughly dried. The same attachment used during sweat collection is used to keep the absorbent disk flat against the skin. After 10 minutes, the absorbent disk is removed and weighed again. The difference in weights is converted to volume of water using the density of water (1 g/mL). The volume collected over the duration of collection yields the sweat rate.

### Experiments

For all experiments, participants are asked to fast for at least 8 hours prior to the start of the experiment and throughout the entire duration. All experiments are started in the morning at approximately 9AM. The custom testing device is fixed to the subject’s volar forearm approximately 15 cm proximal to the distal wrist crease. Sweat production is then stimulated as described above. After a 20-min wait period, the two experiments described below begin.

#### Glucose flux under normal conditions

Glucose flux under normal conditions was measured using the following procedure (S6 Fig). Sweat rate is measured gravimetrically for 10 mins. Midway through the sweat rate measurement, blood glucose is measured using the fingerstick test described above. Immediately after the sweat rate measurement completes, sweat is collected for 45 mins as described above. This procedure of measuring sweat rate, blood glucose, and collecting sweat is repeated 6–8 times. The experiment ends with a final sweat rate and blood glucose measurement. Both sweat rate and blood glucose measurements are averaged from before and after each collection period to determine the average sweat rate and blood glucose during the collection period. The sweat glucose concentration during the collection period is determined using the sweat analysis procedure described above.

#### Glucose flux under electroosmotic flow and paracellular permeability enhancement with electroosmotic flow

The same procedure of measuring sweat rate, blood glucose, and collecting sweat is repeated for 8, 20-min collection periods: two before treatment, four with treatment, and two after treatment (S7 Fig). The treatment was iontophoresis of either acetate which should induce only electroosmotic flow or citrate which should induce both paracellular permeability enhancement and electroosmotic flow. Simply put—acetate is a buffer but not a chelator, citrate is both a buffer and a chelator. The absorbent collection disks were pre-wetted with 30 μL of either a 500 mM acetate (pH 5.5) or citrate (pH 6.5) solution (Sigma-Aldrich). Then, the pre-wetted disk was placed into the testing device and pressed against the testing area using a conductive attachment. Using the same counter electrode used for sweat stimulation, a lab-grade potentiostat drives the acetate or citrate into the skin by applying a constant current density of 0.04 mA/cm^2^ for the entire 20-min collection period. For the two collection periods before and after treatment, the absorbent disks were still pre-wetted, but no current was applied. It is important to note, that the 0.04 mA/cm^2^ utilized here is >10X less than the current density utilized with the GlucoWatch Biographer, and further optimization of current density in future work might show even greater increases in glucose flux.

### IRB protocol and conflict of interest statement

All experiments conducted during this study adhered to a specific study protocol (#2016–4769) that was approved by the University of Cincinnati’s Institutional Review Board (IRB). This study involved 6 healthy participants (5 male, 1 female) between approximately 20 to 40 yrs. of age who were enrolled between Oct. 2016 –Oct. 2017. Participants were recruited via word-of-mouth convenience sampling on University of Cincinnati’s main campus. Participants gave both verbal and written informed consent after receiving both verbal and written explanations of the experiment. All consented individuals completed the study.

Co-author Jason Heikenfeld has an equity interest in Eccrine Systems, Inc., a company that may potentially benefit from the research results, and also serves on the company’s Board. The terms of this arrangement have been reviewed and approved by the University of Cincinnati in accordance with its conflict of interest policies.

### Statistical analysis

Since each participant experienced both treatment types but on separate days and on separate arms, paired t-tests were used to examine the difference between acetate and citrate at each time point. Pairwise comparisons between time points of the same treatment (acetate or citrate) were conducted with a conservative Bonferroni correction.

## Results and discussion

**Fig 2:**
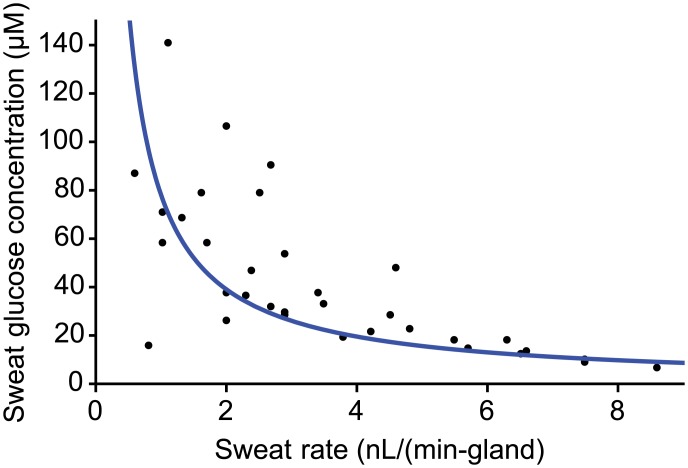
Glucose flux under electroosmotic flow and paracellular permeability enhancement with electroosmotic flow. Total glucose flux measured before, during, and after treatments: iontophoresis of either acetate (black) which should induce only electroosmosis, or citrate (blue) which should induce both paracellular permeability enhancement and electroosmosis (*n* = 4).

For our study, participants were artificially stimulated to sweat. Then, sweat samples were collected from the same site on the forearm for eight time points: two before treatment, four with treatment, and two after treatment. The treatment consisted of RI of either acetate (control), which should induce only electroosmotic flow, or citrate, which should induce both paracellular permeability enhancement and electroosmotic flow. Sweat rate and blood glucose were measured for each collected sample. Total glucose flux—the product of the measured sweat glucose concentration and the measured sweat rate—is plotted for each condition (Fig 2).

Citrate produced significantly higher glucose fluxes compared to acetate at both the final treatment period (6) and the first recovery time point (7), *p* < 0.05. No significant differences exist at other time points. This suggests that the paracellular permeability enhancement induced by the delivery of citrate takes time to develop and once developed takes time to recover.

No significant differences exist between any of the acetate time points. This suggests that the glucose flux is not improved by electroosmotic flow alone. However, with citrate, the second to last and final treatment time points (5 and 6) were each significantly different compared to both of the initial baseline time points (1 and 2), *p* < 0.05. In addition, the final treatment time point (6) was significantly different from the final post-treatment baseline time point (8), *p* < 0.05. This again suggests that it takes time for the paracellular permeability enhancement to develop and once developed the recovery is not instantaneous.

**Fig 3:**
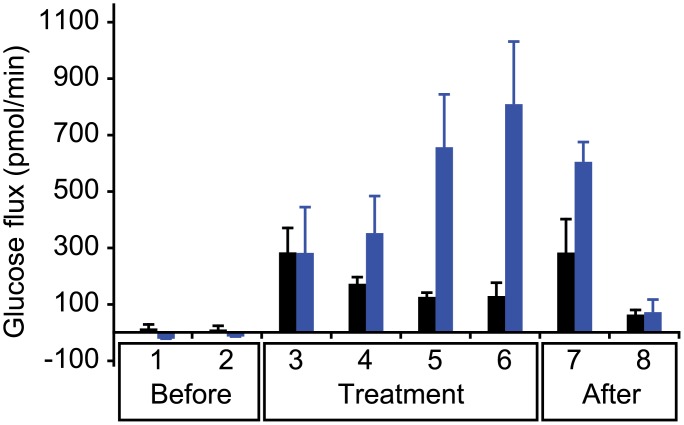
Glucose flux under normal conditions. Sweat glucose concentration and sweat rates measured at various time points following artificial sweat stimulation. Sweat glucose concentration is inversely proportional to sweat rate. The average glucose flux for all participants is roughly 100 fmol/(min-gland) (*n* = 6).

Participants were again artificially stimulated to sweat and developed a peak sweating response that gradually decayed over the course of several hours [22]. Sweat glucose concentrations and sweat rates were measured at various time points during this decay on the same site on the forearm (Fig 3). As sweat rate increases, sweat glucose concentration decreases. This sweat rate dependence exists because glucose flux is largely independent of sweat rate. In fact, all participants had similar glucose fluxes: ~100 fmol/(min-gland) (Fig 3). Since participants were asked to fast prior to the study, their resting blood glucose values were both steady and similar, making the only difference the permeability of the sweat gland. This implies that a basal sweat gland permeability may exist and that high sweat rates simply dilute the concentration. However, since basal glucose permeability was only measured from a total of six male and female participants, it is difficult to discern the impact of demographic factors, such as age or sex. Future work should include a larger, more diverse sample set.

## Conclusions

Since the goal of sweat biosensing is to measure the concentrations of biomarkers in blood or ISF, there must exist a way to relate the measured concentration in sweat to that of blood or ISF. While this may be possible for normal sweat collection conditions, RI with chelators provides additional routes that must be considered when attempting correlate sweat and blood concentrations: passive paracellular diffusion, and electroosmotic flow of biomarker-rich ISF (see S1 Text for equation-driven description). Paracellular permeability enhancement increases both routes making it difficult to determine the extent of increased paracellular permeability vs increased fraction of ISF in sweat. One possible work-around is a ratio-metric approach that compares the sweat concentration of a target biomarker to another biomarker which is relatively stable in blood or ISF. For example, the sweat cytokine to albumin ratio may allow better correlation with blood cytokines. Future work should be focused on resolving this correlation issue.

This study also represents a potential paradigm shift in the way biomarkers in sweat are analyzed by taking a *flux*-based rather than *concentration*-based approach. The fundamental flaw with focusing on concentration is that it is dependent on the total water flux. Previous sweat biomarker studies ignored the importance of measuring sweat rate when measuring the concentration of biomarkers [3,4,30]. By focusing on biomarker flux, the effect of sweat rate on the concentration can be normalized. Under normal sweat collection conditions, this normalization is simple; the product of sweat rate and concentration yields biomarker flux. If both the flux and the permeability of the sweat gland is known, then the biomarker’s concentration in blood or ISF can be calculated.

This study represents the first step in the development of permeability enhancement with electroosmosis. Not only does this method increase glucose flux in sweat, and thereby concentration, but it also holds substantial promise for increasing flux and concentration of other dilute biomarkers, making them easier to detect with currently available biosensors.

## Supporting information

S1 Dataset Raw data for Figs 2 and 3. (XLSX)

S1 Fig Custom device. Custom two-part device for sweat tests is shown with base and multiple twist-lock attachments.
(PNG)

S2 Fig Natural vs artificial sweat stimulation. Natural stimulation occurs in response to thermal loading or metabolic activity which triggers the release of acetylcholine. Artificial stimulation is possible via iontophoresis of a positively-charged acetylcholine look alike, carbachol. (Credit: Microchip Technology Inc.).
(PNG)

S3 Fig Flow diagram of sweat stimulation. First, the skin is cleaned with water and isopropyl alcohol. The base of the two-part device is secured with adhesive and strapped to the forearm of a subject. A carbachol-containing drug disk is loaded and a conductive attachment is locked into place. A constant current is then applied to iontophoretically deliver the sweat-stimulating drug.
(PNG)

S4 Fig Flow diagram of sweat collection and analysis. Petroleum jelly is applied to the testing area to form a barrier between sweat and epidermal contaminants. An absorbent, sweat collection disk is placed onto the testing area. A screwcap will be placed to prevent evaporation during collection. Sweat collection disk is spun down to collect liquid sample. Liquid sweat sample is analyzed using standard assays.
(PNG)

S5 Fig Flow diagram of sweat rate measurements. Absorbent disks are weighed prior to being placed on the skin. After 10 minutes, the disks are reweighed. The difference in weights over time provides the sweat rate.
(PNG)

S6 Fig Block diagram of experiment: Glucose flux under normal conditions. (PNG)

S7 Fig Block diagram of experiment: Glucose flux under electroosmotic flow and paracellular permeability enhancement with electroosmotic flow. (PNG)

S1 Text Sweat/Blood glucose correlation discussion. Equation-driven discussion regarding how measured sweat glucose concentrations correlate to blood glucose concentrations.
(DOCX)

## References

[1] Sonner Z, Wilder E, Heikenfeld J, Kasting G, Beyette F, Swaile D, et al The microfluidics of the eccrine sweat gland, including biomarker partitioning, transport, and biosensing implications. Biomicrofluidics. 2015;9: 031301 10.1063/1.4921039 26045728 PMC4433483

[2] Anderson JM, Van Itallie CM, Van Itallie CM. Physiology and Function of the Tight Junction. Cold Spring Harb Perspect Biol. 2009;1: a002584–a002584. 10.1101/cshperspect.a002584 20066090 PMC2742087

[3] Moyer J, Wilson D, Finkelshtein I, Wong B, Potts R. Correlation Between Sweat Glucose and Blood Glucose in Subjects with Diabetes. Diabetes Technol Ther. 2012;14: 398–402. 10.1089/dia.2011.0262 22376082

[4] Lee H, Choi TK, Lee YB, Cho HR, Ghaffari R, Wang L, et al A graphene-based electrochemical device with thermoresponsive microneedles for diabetes monitoring and therapy. Nat Nanotechnol. Nature Publishing Group; 2016;11: 1–30. 10.1038/nnano.2016.38 26999482

[5] Heikenfeld J. Non-invasive Analyte Access and Sensing through Eccrine Sweat: Challenges and Outlook circa 2016. Electroanalysis. 2016;28: 1242–1249. 10.1002/elan.201600018

[6] Tsukita S, Furuse M, Itoh M. Multifunctional strands in tight junctions. Nat Rev Mol Cell Biol. 2001;2: 285–293. 10.1038/35067088 11283726

[7] Steed E, Balda MS, Matter K. Dynamics and functions of tight junctions. Trends Cell Biol. Elsevier Ltd; 2010;20: 142–9. 10.1016/j.tcb.2009.12.002 20061152

[8] Lindmark T, Kimura Y A P, Lindmark T, Kimura Y, Artursson P. Absorption enhancement through intracellular regulation of tight junction permeability by medium chain fatty acids in Caco-2 cells. J Pharmacol Exp Ther. 1998;284: 362–9. Available: http://www.ncbi.nlm.nih.gov/pubmed/9435199 9435199

[9] Tomita M, Hayashi M, Awazu S. Absorption-Enhancing Mechanism of EDTA, Caprate, and Decanoylcarnitine in Caco-2 Cells. J Pharm Sci. 1996;85: 608–611. 10.1021/js9504604 8773957

[10] Ghartey-Tagoe EB, Morgan JS, Neish AS, Prausnitz MR. Increased permeability of intestinal epithelial monolayers mediated by electroporation. J Control Release. 2005;103: 177–190. 10.1016/j.jconrel.2004.11.021 15710509

[11] Salama NN, Eddington ND, Fasano A. Tight junction modulation and its relationship to drug delivery. Adv Drug Deliv Rev. 2006;58: 15–28. 10.1016/j.addr.2006.01.003 16517003

[12] Deli MA. Potential use of tight junction modulators to reversibly open membranous barriers and improve drug delivery. Biochim Biophys Acta—Biomembr. Elsevier B.V.; 2009;1788: 892–910. 10.1016/j.bbamem.2008.09.016 18983815

[13] Gonzalez-Mariscal L, Contreras RG, Bolívar JJ, Ponce A, Chávez De Ramirez B, Cereijido M. Role of calcium in tight junction formation between epithelial cells. Am J Physiol. 1990;259: C978–86. Available: http://eutils.ncbi.nlm.nih.gov/entrez/eutils/elink.fcgi?dbfrom=pubmed&id=2124417&retmode=ref&cmd=prlinks%5Cnpapers3://publication/uuid/71CC224E-FA05-4AC4-8C02-8FB69843086D 10.1152/ajpcell.1990.259.6.C978 2124417

[14] Bhat M, Toledo-Velasquez D, Wang L, Malanga CJ, Ma JK, Rojanasakul Y. Regulation of tight junction permeability by calcium mediators and cell cytoskeleton in rabbit tracheal epithelium. Pharm Res. 1993;10: 991–7. 10.1023/A:1018906504944 8378262

[15] González-Mariscal L, Tapia R, Chamorro D. Crosstalk of tight junction components with signaling pathways. Biochim Biophys Acta—Biomembr. 2008;1778: 729–756. 10.1016/j.bbamem.2007.08.018 17950242

[16] Ivanov AI. Endocytosis of Epithelial Apical Junctional Proteins by a Clathrin-mediated Pathway into a Unique Storage Compartment. Mol Biol Cell. 2003;15: 176–188. 10.1091/mbc.E03-05-0319 14528017 PMC307538

[17] Leboulanger B, Guy RH, Delgado-Charro MB. Reverse iontophoresis for non-invasive transdermal monitoring. Physiol Meas. 2004;25: R35–R50. 10.1088/0967-3334/25/3/R01 15253111

[18] Tierney MJ, Tamada JA, Potts RO, Jovanovic L, Garg S. Clinical evaluation of the GlucoWatch^®^ biographer: a continual, non-invasive glucose monitor for patients with diabetes. Biosens Bioelectron. 2001;16: 621–629. 10.1016/S0956-5663(01)00189-0 11679237

[19] Potts RO, Tamada JA., Tierney MA. Glucose monitoring by reverse iontophoresis. Diabetes Metab Res Rev. 2002;18: S49–S53. 10.1002/dmrr.210 11921430

[20] Tierney MJ, Kim HL, Burns MD, Tamada JA, Potts RO. Electroanalysis of Glucose in Transcutaneously Extracted Samples. Electroanalysis. 2000;12: 666–671. 10.1002/1521-4109(200005)12:9<666::AID-ELAN666>3.0.CO;2-C

[21] Fiume MM, Heldreth BA, Bergfeld WF, Belsito D V, Hill RA, Klaassen CD, et al Safety Assessment of Citric Acid, Inorganic Citrate Salts, and Alkyl Citrate Esters as Used in Cosmetics. Int J Toxicol. 2014;33: 16S–46S. 10.1177/1091581814526891 24861367

[22] Simmers P, Li SK, Kasting G, Heikenfeld J. Prolonged and localized sweat stimulation by iontophoretic delivery of the slowly-metabolized cholinergic agent carbachol. J Dermatol Sci. Japanese Society for Investigative Dermatology; 2018;89: 40–51. 10.1016/j.jdermsci.2017.10.013 29128285

[23] Grice EA, Segre JA. The skin microbiome. Nat Rev Microbiol. 2011;9: 244–253. 10.1038/nrmicro2537 21407241 PMC3535073

[24] Davis CP. Normal Flora [Internet]. Medical Microbiology. University of Texas Medical Branch at Galveston; 1996. NBK7617 [bookaccession] 21413249

[25] Sender R, Fuchs S, Milo R. Revised Estimates for the Number of Human and Bacteria Cells in the Body. PLoS Biol. Blackwell Publishing company; 2016;14: e1002533 10.1371/journal.pbio.1002533 27541692 PMC4991899

[26] Liu G, Ho C, Slappey N, Zhou Z, Snelgrove SEE, Brown M, et al A wearable conductivity sensor for wireless real-time sweat monitoring. Sensors Actuators, B Chem. 2016;227: 35–42. 10.1016/j.snb.2015.12.034

[27] Ely MR, Kenefick RW, Cheuvront SN, Chinevere TD, Lacher CP, Lukaski HC, et al Surface contamination artificially elevates initial sweat mineral concentrations. J Appl Physiol. 2011;110: 1534–1540. 10.1152/japplphysiol.01437.2010 21512152

[28] Sheu HM, Chao SC, Wong TW, Yu-Yun Lee J, Tsai JC. Human skin surface lipid film: an ultrastructural study and interaction with corneocytes and intercellular lipid lamellae of the stratum corneum. Br J Dermatol. Blackwell Science Ltd; 1999;140: 385–91. 10.1046/j.1365-2133.1999.02697.x 10233255

[29] Cork MJ, Robinson DA, Vasilopoulos Y, Ferguson A, Moustafa M, MacGowan A, et al New perspectives on epidermal barrier dysfunction in atopic dermatitis: Gene–environment interactions. J Allergy Clin Immunol. 2006;118: 3–21. 10.1016/j.jaci.2006.04.042 16815133

[30] Boysen TC, Yanagawa S, Sato F, Sato K. A modified anaerobic method of sweat collection. J Appl Physiol. American Physiological Society; 1984;56: 1302–7. 10.1152/jappl.1984.56.5.1302 6327585

[31] Peng R, Sonner Z, Hauke A, Wilder E, Kasting J, Gaillard T, et al A new oil/membrane approach for integrated sweat sampling and sensing: sample volumes reduced from μL’s to nL’s and reduction of analyte contamination from skin. Lab Chip. The Royal Society of Chemistry; 2016;16: 4415–4423. 10.1039/c6lc01013j 27752680

[32] Cha KH, Jensen GC, Balijepalli AS, Cohan BE, Meyerhoff ME. Evaluation of Commercial Glucometer Test Strips for Potential Measurement of Glucose in Tears. Anal Chem. 2014;86: 1902–1908. 10.1021/ac4040168 24428813

