# Supplementary figures and images for: Enhancing glucose flux into sweat by increasing paracellular permeability of the sweat gland
Source: PLoS One. 2018 Jul 16;13(7):e0200009. doi: 10.1371/journal.pone.0200009 (PMC6047769; doi:10.1371/journal.pone.0200009)

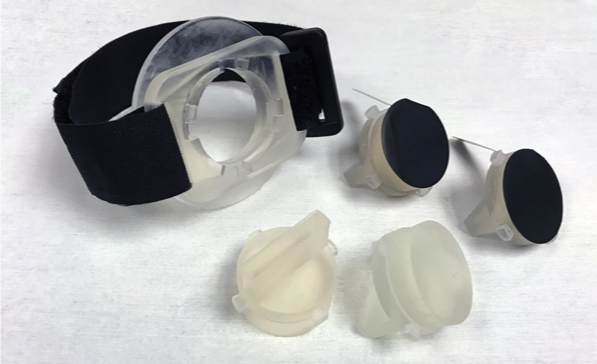

Supplement: S1 Fig — Custom two-part device for sweat tests is shown with base and multiple twist-lock attachments. (PNG) [file pone.0200009.s002.png]

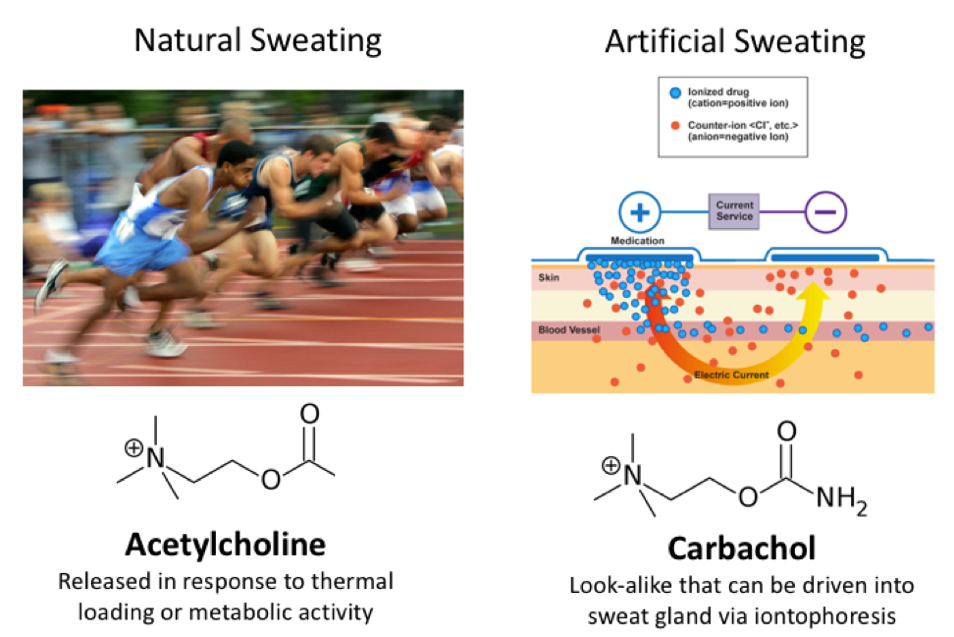

Supplement: S2 Fig — Natural stimulation occurs in response to thermal loading or metabolic activity which triggers the release of acetylcholine. Artificial stimulation is possible via iontophoresis of a positively-charged acetylcholine look alike, carbachol. (Credit: Microchip Technology Inc.). (PNG) [file pone.0200009.s003.png]

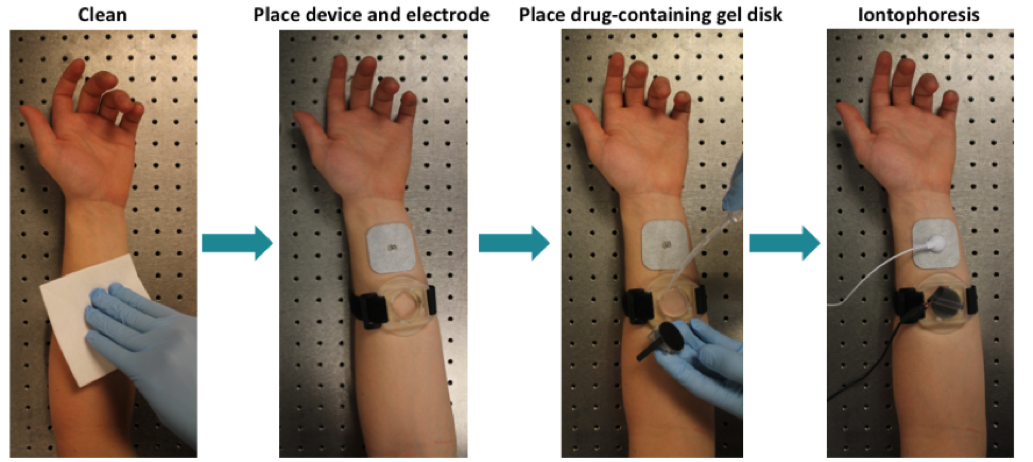

Supplement: S3 Fig — First, the skin is cleaned with water and isopropyl alcohol. The base of the two-part device is secured with adhesive and strapped to the forearm of a subject. A carbachol-containing drug disk is loaded and a conductive attachment is locked into place. A constant current is then applied to iontophoretically deliver the sweat-stimulating drug. (PNG) [file pone.0200009.s004.png]

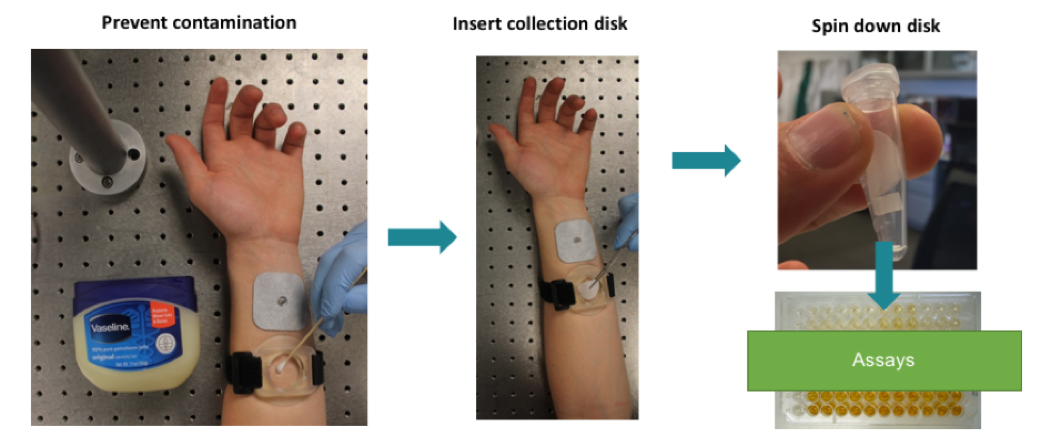

Supplement: S4 Fig — Petroleum jelly is applied to the testing area to form a barrier between sweat and epidermal contaminants. An absorbent, sweat collection disk is placed onto the testing area. A screwcap will be placed to prevent evaporation during collection. Sweat collection disk is spun down to collect liquid sample. Liquid sweat sample is analyzed using standard assays. (PNG) [file pone.0200009.s005.png]

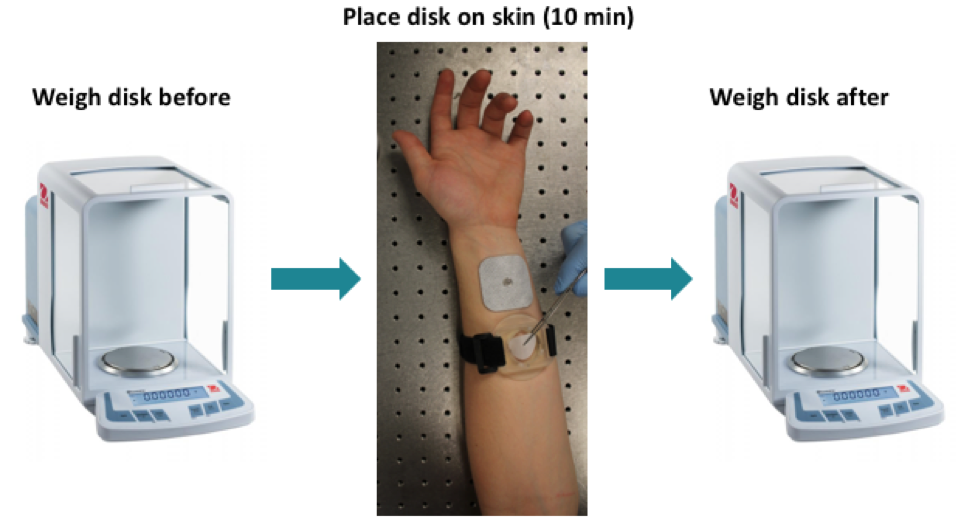

Supplement: S5 Fig — Absorbent disks are weighed prior to being placed on the skin. After 10 minutes, the disks are reweighed. The difference in weights over time provides the sweat rate. (PNG) [file pone.0200009.s006.png]

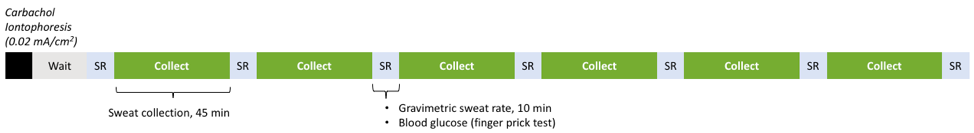

Supplement: S6 Fig — (PNG) [file pone.0200009.s007.png]

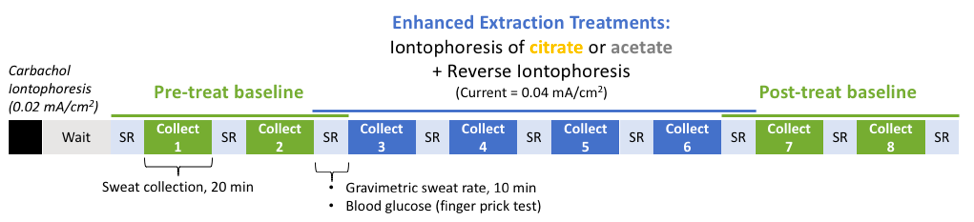

Supplement: S7 Fig — (PNG) [file pone.0200009.s008.png]
