# Supplementary material for: Enhancing glucose flux into sweat by increasing paracellular permeability of the sweat gland
Source: PLoS One. 2018 Jul 16;13(7):e0200009. doi: 10.1371/journal.pone.0200009 (PMC6047769; doi:10.1371/journal.pone.0200009)
Supplement: S1 Text — Equation-driven discussion regarding how measured sweat glucose concentrations correlate to blood glucose concentrations. (DOCX) [file pone.0200009.s009.docx]

**S1** **Text**

Under normal conditions, glucose flux into sweat is driven by Fick’s diffusion:

$$\boldsymbol{Eq. 1:} {Flux}_{Fick's}=p(\Delta c)$$

where flux is proportional to the concentration gradient ($\Delta c$) with a proportionality constant, or permeability constant ($p$). The glucose concentration in sweat is much lower than that in blood or ISF, and so the concentration gradient can be simplified.

$$\boldsymbol{Eq. 2:}\Delta c=c_{blood/ISF}-c_{sweat}\approx c_{blood/ISF}$$

where $c_{blood/ISF}$is the concentration of glucose in blood or ISF and $c_{sweat}$ is the concentration of glucose in sweat. The sweat glucose concentration is simply the total glucose flux (Fick’s diffusion) over the total water flux (sweat rate).

$$\boldsymbol{Eq. 3:}c_{sweat} =\frac{{Flux}_{Fick's}}{Q_{sweat}}=\frac{p\left( c_{blood/ISF} \right)}{Q_{sweat}}$$

Therefore, fitting an inverse function to the sweat glucose concentration and sweat rate relationship yields glucose flux.

Now with an understanding of the total glucose flux under normal conditions, we examined the total glucose flux in the presence of either electroosmotic flow or paracellular permeability enhancement with electroosmotic flow. In these cases, both the total glucose flux and total water flux are more complicated than in Eq. 3. There are two routes of entry for fluid into sweat—natural sweating ($Q_{sweat}$) and active flow of ISF into the gland ($Q_{ISF}$). Natural sweating brings water into the sweat lumen via aquaporins in the secretory coil ^1^. Assuming a moderate sweat rate and sweat gland density for the forearm, the flow of water due to natural sweating ($Q_{sweat}$) is ~500 nL/min-cm^2^.

$${\boldsymbol{Eq. 4:} Q}_{sweat}=\left( 5 \frac{nL}{min-gland} \right)\left( 100 \frac{glands}{{cm}^{2}} \right)=500\frac{nL}{min-{cm}^{2}}$$

Alternatively, electroosmosis results in active flow of biomarker-rich ISF into the sweat gland ($Q_{ISF}$). Using an average value from literature for the volume per charge relationship ^2^ and the current density selected for this study (0.04 mA/cm^2^), the flow of ISF into sweat ($Q_{ISF}$) is calculated to be only ~6 nL/min-cm^2^.

$${\boldsymbol{Eq. 5:} Q}_{ISF}=\left( 150 \frac{nL}{mA-min} \right)\left( 0.04 \frac{mA}{{cm}^{2}} \right)=6\frac{nL}{min-{cm}^{2}}$$

Because $Q_{ISF}$ is much smaller than $Q_{sweat}$, its contribution to the total water flux can be ignored. However, its contribution to glucose flux *is* significant, as the ISF that passes into sweat is biomarker-rich. The glucose flux due to electroosmosis of ISF (${Flux}_{ISF}$) is proportional to the flow of ISF ($Q_{ISF}$), the concentration of glucose in ISF ($c_{ISF}$), and a proportionality constant ($k$) that represents some resistance to glucose diffusion by the paracellular pathway.

$$\boldsymbol{Eq. 6:} {Flux}_{ISF}=k(c_{ISF})\left( Q_{ISF} \right)$$

Taken together, the following is a constitutive equation that describes all three experimental cases: normal ($p=p_{normal}$; $k\approx0$), electroosmotic flow ($p=p_{normal}$; $k>0$), and paracellular permeability enhancement with electroosmotic flow ($p>p_{normal}$; $k\gg0$).

$$\boldsymbol{Eq. 7:}c_{sweat} =\frac{{Flux}_{Fick^{'}s}+{Flux}_{ISF}}{Q_{sweat}+Q_{ISF}}=\frac{p\left( \Delta c \right)+k\left( c_{ISF} \right)\left( Q_{ISF} \right)}{Q_{sweat}+Q_{ISF}}\approx\frac{p\left( c_{blood/ISF} \right)+k(c_{blood/ISF})\left( Q_{ISF} \right)}{Q_{sweat}}$$

Rewritten, the following equation shows that the product of the measured sweat glucose concentration ($c_{sweat}$) and the measured sweat rate ($Q_{sweat})$yields total glucose flux (${Flux}_{Fick^{'}s}+{Flux}_{ISF}$).

$$\boldsymbol{Eq. 8:}c_{sweat} \left( Q_{sweat} \right)={Flux}_{Fick^{'}s}+{Flux}_{ISF}=p\left( \Delta c \right)+k\left( c_{ISF} \right)\left( Q_{ISF} \right)\approx p\left( c_{blood/ISF} \right)+k(c_{blood/ISF})\left( Q_{ISF} \right)$$

**References**

1 Bovell D. The human eccrine sweat gland: Structure, function and disorders. *J Local Glob Heal Sci* 2015;**2015**:5. https://doi.org/10.5339/jlghs.2015.5.

2 Pikal MJ, Shah S. Transport mechanisms in iontophoresis. III. An experimental study of the contributions of electroosmotic flow and permeability change in transport of low and high molecular weight solutes. *Pharm Res* 1990;**7**:222–9. https://doi.org/10.1023/A:1015809725688.
